# Circular RNA vaccine induces potent T cell responses

**DOI:** 10.1073/pnas.2302191120

**Published:** 2023-05-08

**Authors:** Laura Amaya, Lilit Grigoryan, Zhijian Li, Audrey Lee, Paul A. Wender, Bali Pulendran, Howard Y. Chang

**Affiliations:** ^a^Center for Personal Dynamic Regulomes, Stanford University, Stanford, CA 94305; ^b^Institute for Stem Cell Biology and Regenerative Medicine, Stanford University School of Medicine, Stanford, CA 94305; ^c^Institute for Immunity, Transplantation and Infection, Stanford University, Stanford, CA 94305; ^d^Department of Chemistry, Stanford University, Stanford, CA 94305; ^e^Department of Chemical and Systems Biology, Stanford University, Stanford, CA 94305; ^f^HHMI, Stanford University, Stanford, CA 94305

**Keywords:** circular RNA, CD8 T cells, vaccine

## Abstract

Circular RNAs (circRNAs) are a class of RNAs commonly found across eukaryotes and viruses, characterized by their resistance to exonuclease-mediated degradation. Their superior stability compared to linear RNAs, combined with previous work showing that engineered circRNAs serve as efficient protein translation templates, make circRNA a promising candidate for RNA medicine. Here, we systematically examine the adjuvant activity, route of administration, and antigen-specific immunity of circRNA vaccination in mice. Potent circRNA adjuvant activity is associated with RNA uptake and activation of myeloid cells in the draining lymph nodes and transient cytokine release. Immunization of mice with engineered circRNA encoding a protein antigen delivered by a charge-altering releasable transporter induced innate activation of dendritic cells, robust antigen-specific CD8 T cell responses in lymph nodes and tissues, and strong antitumor efficacy as a therapeutic cancer vaccine. These results highlight the potential utility of circRNA vaccines for stimulating potent innate and T cell responses in tissues.

## Significance

Circular RNAs (circRNAs) are a unique class of RNAs that are highly stable compared to linear mRNAs and can be engineered to provide durable protein expression. This study demonstrates that circRNA encoding antigenic protein sequences delivered by a charge-altering releasable transporter can effectively serve as both an adjuvant and an immunogen, inducing potent cellular immunity and leading to tumor clearance when used as a therapeutic vaccine. These results suggest engineered circRNAs for the development of vaccines and therapeutics.

Circular RNAs (circRNAs) are covalently closed single-stranded RNA molecules derived from back-splicing processes (1). Most eukaryotic circRNAs are noncoding RNAs with potential regulatory functions in gene expression (2). Among other biochemical functions, circRNAs interact with other noncoding RNAs and serve as microRNA sponges (3), protein scaffolds (4), canonical splicing competitors (5), and protein nuclear translocation triggers (6). However, a small portion of endogenous circRNAs possesses internal ribosome entry sites (IRESs) and can act as protein templates, inducing translation in a cap-independent manner (7–9). Large amounts of circRNAs can also be synthesized in vitro through an in vitro transcription reaction (IVT) that includes a DNA template and a phage RNA polymerase (10). Several efforts are being made to improve the circularization and translation efficiency of circRNAs (11, 12). Currently, up to 10 kb transcripts in length can circularize and express open-reading frames (13).

Recent studies have also suggested a potential role of circRNAs as modulators of the immune system. Some endogenous circRNAs can inhibit protein kinase R and restrain innate immunity (14). Changes in circRNA abundance have been associated with the occurrence of autoimmune disease (15) and proposed as potential regulators of tumor immunity (16). The recognition of in vitro-transcribed circRNAs by pattern recognition receptors when delivered into mammalian cells has also been described (17). Differences in the structure and covalent modifications of cellular and pathogenic circRNA distinguish self from nonself (17–19). Chen et al. showed that RIG-I directly senses exogenous circRNA and initiates an innate immune signaling cascade dependent on the absence of *N*6-methyladenosine (m6A) as a mark of self-identity (18).

Due to their covalently closed loop structures and the absence of 5′ caps or 3′ poly A tails, circRNAs are more stable compared to linear RNA. CircRNAs are highly stable in blood (20) and may be released from cells via extracellular vesicles (21). These characteristics have drawn increasing attention to circRNAs for applications in clinical practice as diagnostic and prognostic biomarkers (22, 23), and more recently, as candidates for vaccines. A circRNA-based vaccine was shown to induce broad-spectrum protection against SARS-CoV-2 in nonhuman primates (24). However, the mechanisms behind the in vivo recognition of circRNA, its translation, and activating signals leading to protective immunity remain poorly understood.

Cytotoxic (CD8) T cells represent a major target of vaccination, as CD8 T cell–mediated protection has been shown to be important in both contexts of viral infections and tumor immunity. Heterologous viral vector regimens can uniquely elicit tissue-resident T cells at sites of infection compared to recombinant protein or mRNA vaccines (25, 26), but the former is difficult to scale and implement in humans. Thus, there is an unmet need to identify additional vaccine platforms to elicit T cell immunity. One challenge is that simultaneous antigen delivery and adjuvant to antigen-presenting cells are required to induce strong T cell immunity in vaccination settings (27). Here, we propose simplifying the formulation of RNA vaccines by using circRNA as both immunogen and adjuvant. We previously found that in vitro-transcribed circRNAs, which are efficiently circularized via the self-splicing Td intron from T4 bacteriophage, are highly immunogenic in vivo (17, 18). Using circRNA as an adjuvant elicited potent B and T cell responses even when delivered without an RNA transfection reagent (18). However, it remains unclear whether exogenous circular RNA encoding antigen could induce antigen-specific immune responses.

In this study, we explore the principles of how exogenous circRNAs interact with the mammalian immune system. We investigate the innate immune responses induced by naked circRNA and circRNA encapsulated in charge-altering releasable transporters (CARTs). Furthermore, we elaborate on the adjuvant properties of circRNA by examining T cell responses induced by different immunization routes and comparing it to commonly used vaccine adjuvants. Finally, we show that immunization with CART-encapsulated circRNA encoding antigen results in successful antigen presentation, induction of potent cellular immunity, and tumor clearance when used as a therapeutic cancer vaccine.

## Results

### CircRNA Acts as a Potent Vaccine Adjuvant in Multiple Immunization Routes When Combined with Soluble Protein.

Adjuvants are essential to improve the effectiveness of vaccines by increasing the strength and duration of the immune response through different modes of action. We previously showed that exogenous circRNA help induce antigen-specific antibodies and T cells when delivered in vivo with soluble protein (18). Here, we further evaluate the potential of circRNA as a unique adjuvant in vaccine formulations. We systematically measured the acute and memory immune responses after vaccination with circRNA compared to commonly used adjuvants.

**Fig. 1.**
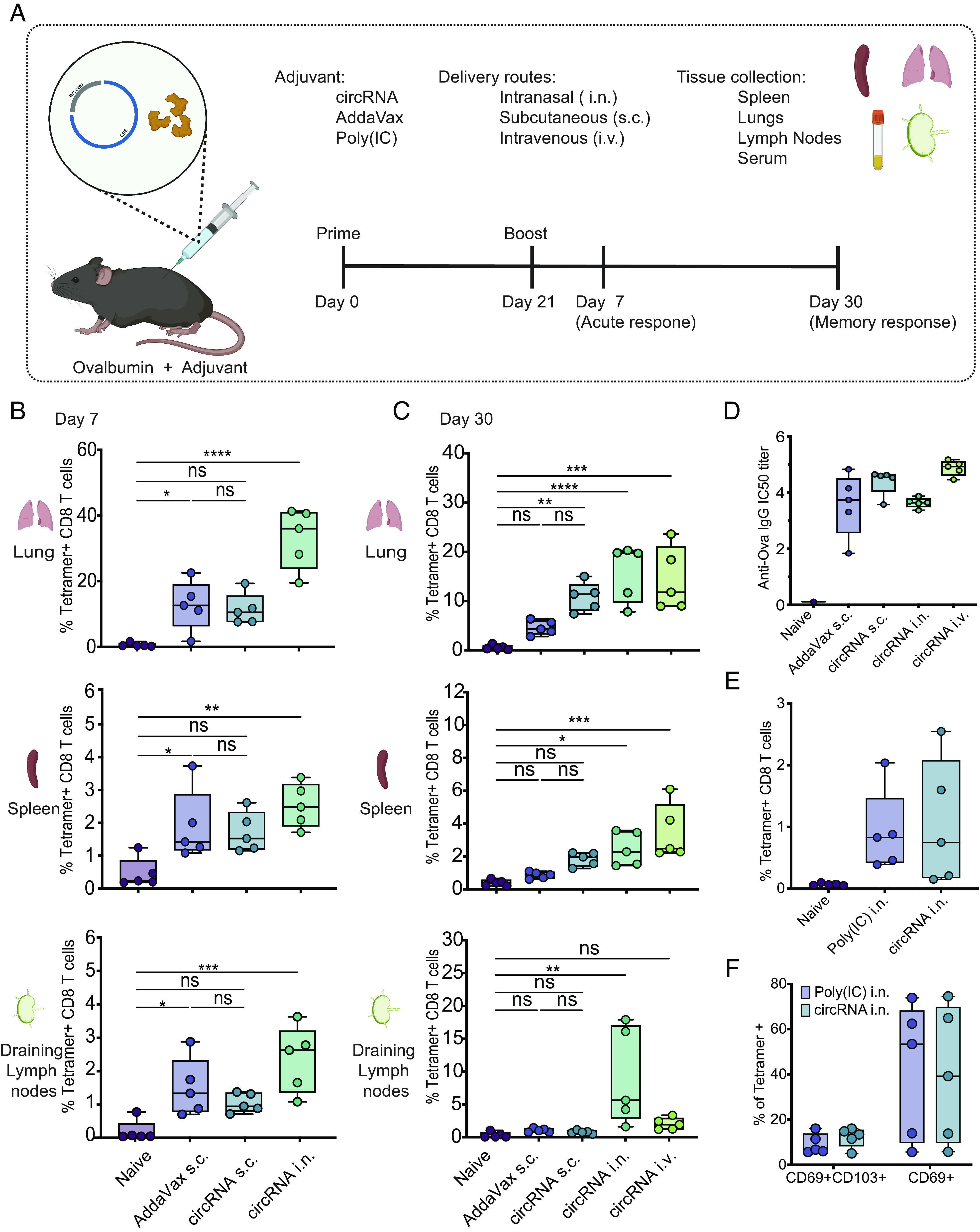
Adjuvant effect of circRNA by different routes of delivery. (*A*) Schematic representation of circRNA immunization strategy via different delivery routes and monitoring of immune responses. OVA protein (50 μg) and circRNA (25 μg), AddaVax (50 μL), or Poly(IC) (25 μg) was delivered by intranasal (i.n.), subcutaneous (s.c.), or intravenous (i.v.) injection. Serum, lung, lymph nodes, and spleen were analyzed at days 7 and 30 postboost. Percentage of OVA-specific T cell responses in lung, spleen, and lymph nodes after (*B*) 7 d or (*C*) 30 d postboost (n = 5, bars represent Min and Max). (*D*) Anti-OVA IgG antibodies in serum measured by ELISA at day 30 postboost after circRNA immunization by different delivery routes (n = 5, bars represent Min and Max). (*E*) Frequency of class I tetramer+ CD8 T cells at day 30 postboost of i.n. delivery of circRNA compared to Poly(IC) (n = 5, bars represent Min and Max). (*F*) Frequency of CD69+ and CD69+CD103+ CD8 TRM in the lungs at day 30 postboost (as percentage of antigen-specific CD8 T cells) (n = 5, bars represent Min and Max). One-way ANOVA was applied in *B*–*E*, and two-way ANOVA in *F*. *P < 0.05, **P < 0.01, ***P < 0.001, ****P < 0.0001. Differences between groups were considered significant for *P* values < 0.05. ns, not significant.

The route of immunization can shape the immune response by determining which antigen-presenting cells (APCs) are activated and where the immune response is focused. Different routes may have different advantages and disadvantages depending on the type of vaccine. Therefore, we immunized C57BL/6 mice with in vitro-synthesized circRNA and chick Ovalbumin protein (referred as OVAp) and compared the magnitude of T cell and antibody responses with three delivery strategies: subcutaneous (s.c), intranasal (i.n.), and intravenous (i.v.). We measured the T cell and antibody responses in the spleen, draining lymph nodes (LNs), and lungs at day 7 and day 30 postboost (Fig. 1*A*). The immune responses observed with immunogenic circRNA [lacking m6A modification (18)] were compared to the results induced by common vaccine adjuvants: AddaVax, a squalene-based oil-in-water nanoemulsion and effective subcutaneous adjuvant (28); and Poly(I:C), a synthetic dsRNA and effective intranasal adjuvant (29).

At day 7 postboost, we observed that subcutaneous injection of circRNA+OVAp induced comparable T cell responses to AddaVax+OVAp in the lungs, spleens, and LNs (as measured by the frequency of MHC class I tetramer+ CD8 T cells) (Fig. 1*B*). Intranasal inoculation of circRNA+OVAp induced the highest T cell responses in the lungs at day 7 postboost, with the frequencies of tetramer-positive cells as high as 40% (Fig. 1*B*). Subcutaneous delivery of circRNA+OVAp induced a twofold higher frequency of antigen-specific CD8 T cells (~10%) compared to AddaVax+OVAp (~5%) at day 30 postboost (Fig. 1*C*), suggesting a potentially enhanced memory T cell induction by circRNA compared to AddaVax. In addition, strong lung memory CD8 T cell responses were observed with the intranasal and intravenous delivery methods (Fig. 1*C*), with the induction of lung-resident memory CD8 T cell (CD69+CD103+) subsets (*SI Appendix*, Fig. S1 *A* and *B*).

To measure the antibody responses following immunization, mice were bled 30 days postboost. We observed similar levels of anti-OVA IgG antibodies in serum among all delivery routes (Fig. 1*D*). However, only intranasal and intravenous immunization induced anti-OVA IgA antibodies in serum (*SI Appendix*, Fig. S1*C*).

In addition, to compare circRNA and Poly(I:C) as intranasal adjuvants, mice were immunized with either adjuvant in combination with soluble OVA protein. CircRNA and Poly(I:C) induced comparable frequencies of antigen-specific CD8 T cells in the lungs at 30 d postboost (Fig. 1*E*), including CD69+ and CD69+CD103+ antigen-specific tissue-resident memory T cells (TRM) (Fig. 1*F*). In addition, circRNA and Poly(I:C) induced similar levels of anti-OVA IgG and IgA antibodies (*SI Appendix*, Fig. S1 *D* and *E*). Taken together, our results suggest that circRNA can be used as a potent vaccine adjuvant in many routes of immunization and induce comparable responses to Poly(I:C) and AddaVax. In addition, we showed that mucosal immunization with circRNA as an adjuvant can induce potent resident memory CD8 T cell (TRM) responses.

### CircRNA Activates Innate Immune Cells When Injected into Mice.

**Fig. 2.**
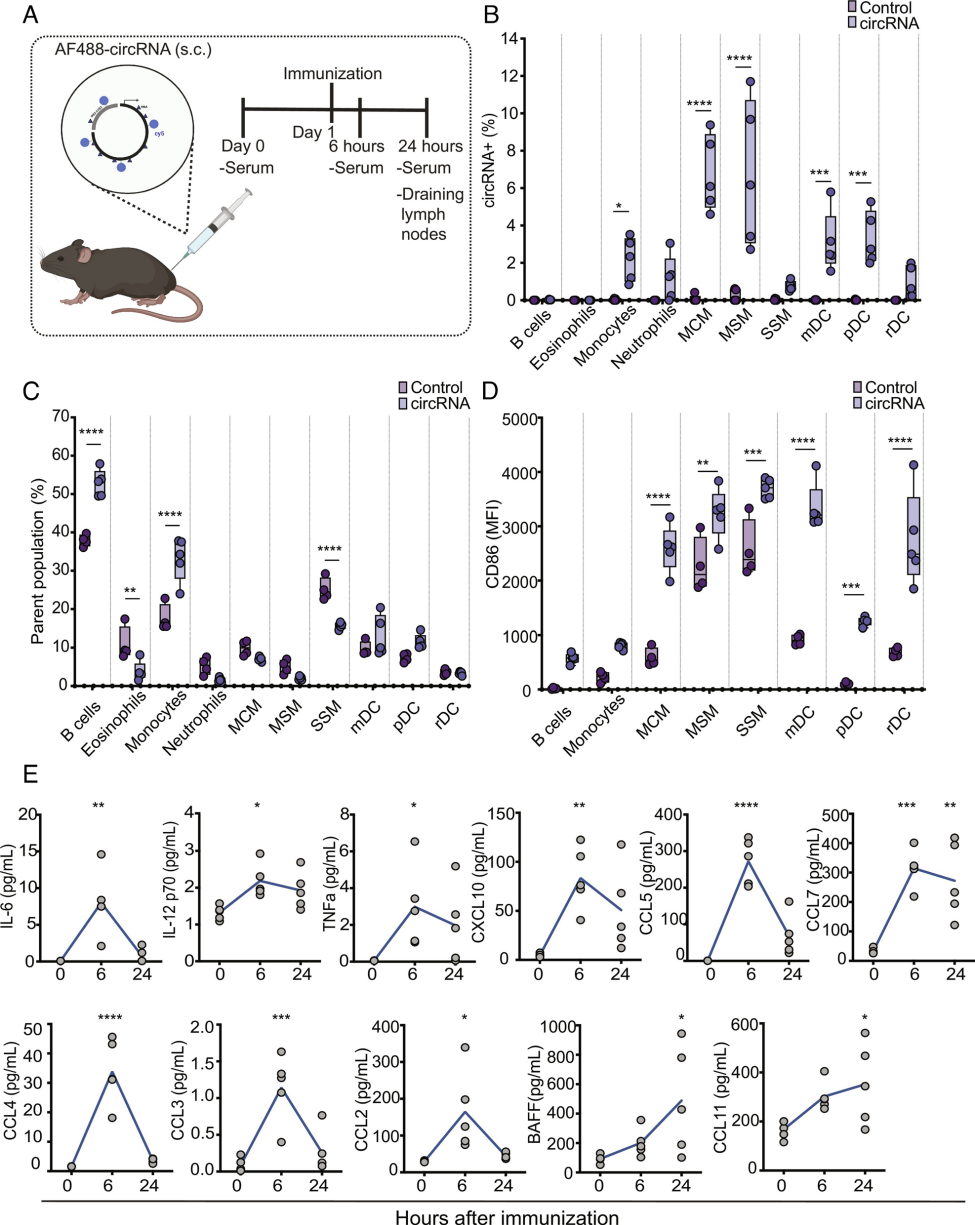
Biodistribution of circRNA and innate recognition. (*A*) Schematic representation of in vivo circRNA delivery and monitoring. Fifty micrograms of AF488-circRNA was delivered subcutaneously, and serum samples were collected 6 and 24 h after delivery. Draining lymph nodes were also analyzed after 24 h by flow cytometry. (*B*) Absolute fraction of fluorescently positive innate cell subsets that take up circRNA (n = 5, bars represent Min and Max). (*C*) Quantification of innate cell subsets proportions and (*D*) fluorescent intensity of activation marker CD86 in distinct innate immune cell subsets in lymph nodes, 24 h after s.c. delivery of fluorescently labeled circRNA (n = 5, bars represent Min and Max). (*E*) Time course analysis of cytokines in serum after circRNA delivery measured by Luminex (n = 5). Two-way ANOVA was applied in *B*–*D*. One-way ANOVA was applied in *E*. *P < 0.05, **P < 0.01, ***P < 0.001, ****P < 0.0001.

The innate immune system plays a fundamental role in programming the adaptive immune response's magnitude, quality, and durability (30). To understand the mechanisms behind the strong adaptive immune responses observed after vaccination with naked circRNA, we surveyed the innate immune compartment for marks of activation and circRNA recognition. We started by determining the biodistribution of circRNAs when delivered in vivo. We conjugated circRNA to the fluorophore AF488 and subcutaneously (s.c.) injected 25 μg of AF488-circRNA into C57BL/6 mice. Serum was analyzed by a Luminex panel of innate cytokines before and after circRNA immunization. In addition, innate immune cell subsets in the draining inguinal lymph nodes (iLNs) were analyzed via flow cytometry at 24 h following immunization (Fig. 2*A*). Innate cell activation was measured by the upregulation of the activation marker CD86 on each cell subset. Monocytes were defined as CD11b+Ly6C+ cells and dendritic cells as CD11c high MHC-II high cells, with DC subsets further subdivided into migratory CD103+ or CD11b+ DCs (mDC) and resident CD8a+ or CD11b+ DCs (rDC). Lymph node (LN) macrophages were identified as CD11b+Ly6CloF4/80+/-CD169+/- and plasmacytoid dendritic cells (pDCs) as CD11b-PDCA-1+ cells. Lastly, neutrophils were defined as CD11b+Ly6G+ and eosinophils as CD11b+Signlec-F+ (*SI Appendix*, Fig. S2*A*).

At 24 h following s.c. injection, circRNA was detected in monocytes, dendritic cells, and several macrophage subsets in the draining lymph nodes. The macrophage subsets taking up circRNAs included marginal cord macrophage (MCMs), marginal sinus macrophage (MSMs), and subcapsular sinus macrophages, (SSM), with MCMs and MSMs having the most significant uptake of circRNA (Fig. 2*B*). Even though no circRNA uptake was observed by B cells in mice, B cell frequencies in the iLNs significantly increased at 24 h post immunization (Fig. 2*C*), and B cell activation, as measured by CD86 upregulation, increased as well (Fig. 2*D*). We observed a significant increase in the frequency (Fig. 2*C*) and activation (Fig. 2*D*) of monocytes in the iLNs, as well as increased activation of all macrophage and dendritic cell subsets compared to untreated controls (Fig. 2*D*).

To examine the serum cytokine response to immunization with circRNA, sera from immunized mice were analyzed at 6- and 24-h postimmunization (*SI Appendix*, Fig. S2*B*). Significant production of chemokines: CCL5, CCL4, CCL3, CCL7, CXCL10, CCL2; and cytokines: IL-6, TNFa, IL-12; was observed, with a peak at 6 h after immunization, followed by a decrease at 24 h (Fig. 2*E*). BAFF and CCL11 showed a continued increase with a peak at 24 h after circRNA immunization (Fig. 2*E*). Taken together, our data suggest that naked circRNA is taken up by innate immune cells when injected into mice and induces subsequent activation of several innate immune cell types while promoting strong induction of inflammatory cytokines.

### CircRNA Induces Immune Activation of Dendritic Cells.

Dendritic cells (DCs) are the most effective antigen-presenting cells, and their maturation indicates the acquisition of several properties, including antigen processing and presentation, migration, and T cell costimulation (31). After observing that dendritic cells are one of the primary innate cell subsets responsible for the recognition of circRNA when delivered in vivo, we next wanted to investigate whether the delivery of naked circRNA would influence the maturation and activation status of dendritic cells. MutuDCs, a murine DC cell line originated from splenic CD8α conventional DC tumors (32), were treated with circRNA or CpG oligodeoxynucleotides, short synthetic single-stranded DNA, known to induce dendritic cell maturation (33). CircRNA induced strong upregulation of costimulatory molecules such as MHC-II, MHC-I, CD80, CD86, and CD40 on MutuDCs, with higher levels of MHC-II and CD40 compared to CpG treatment. (*SI Appendix*, Fig. S3*A*). As innate immune signaling often promotes inflammatory cytokine gene expression to induce DC activation, we next examined cytokine gene expression in MutuDCs after incubation with circRNA. CirRNA treatment significantly induced the expression of proinflammatory cytokine genes IL-1β, TNFa, and IL-6, cytokines required for dendritic cell differentiation and maturation (34) (*SI Appendix*, Fig. S3*B*). CircRNA uptake in MutuDC cells also lead to a significant increase in the mRNA levels of the cytosolic RNA sensors RIG-I and MDA5. This observation in murine dendritic cells indicates that the recognition of circRNA by RIG-I is independent of the cell type, as this effect was previously described in HeLa cells after the transfection of foreign circRNA (17). Flow cytometry results confirmed the upregulation at the protein level of TNFa and IL-6 cytokines in culture supernatant from cells treated with circRNA. Increased levels of MCP1 were also observed. This monocyte chemoattractant protein-1 has the ability to drive the chemotaxis of myeloid and lymphoid cells (35) (*SI Appendix*, Fig. S3*C*). These results indicate that circRNA uptake leads to dendritic cell maturation, which increases the expression of costimulatory molecules and secretion of a wide variety of inflammatory cytokines and chemokines.

### Engineered circRNA-Encoding Protein Leads to Antigen Presentation.

**Fig. 3.**
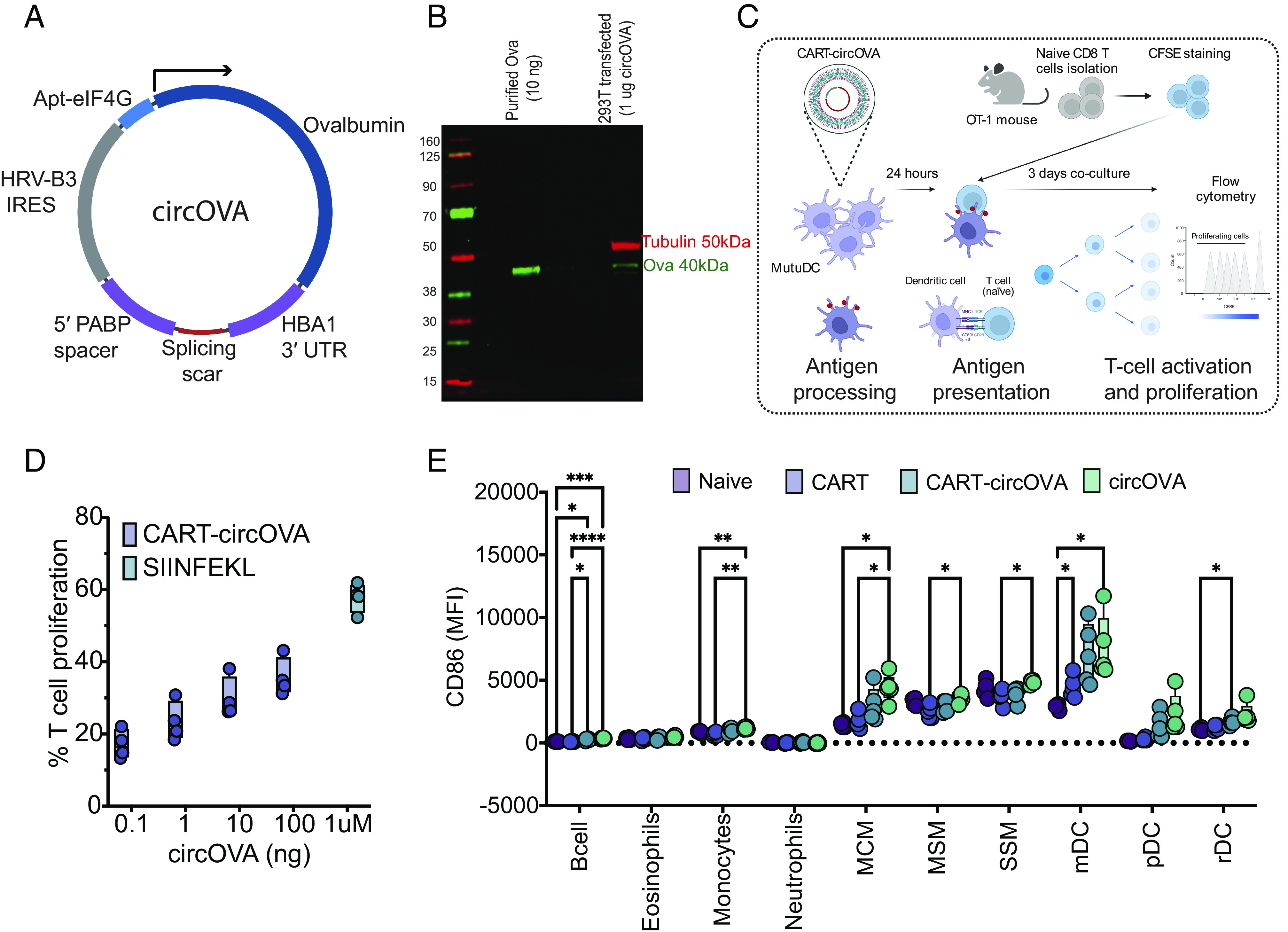
Circular RNA is translated and presented to the immune system. (*A*) Schematic representation of circOVA design and engineered components. Arrow indicates expected open-reading frame. (*B*) Detection of full-length Ovalbumin protein by western blot 24 h after transfection of 293T cells with circOVA. (*C*) Experimental design of proliferation assay used to measure antigen-specific T cell proliferation level of OT-I cells cocultured with MutuDC cells incubated with CART-circOVA. (*D*) CART-circOVA titration to determine the minimum amount required to induce antigen-specific T cell proliferation and peptide SINFEKL as positive control (n = 4, bars represent Min and Max). (*E*) CD86 expression of innate cell subsets 24 h after s.c. delivery of circRNA, circRNA delivered with CART (CART-circOVA), and CART alone (n = 5, bars represent Min and Max). Two-way ANOVA was applied *P < 0.05, **P < 0.01, ***P < 0.001, ****P < 0.0001. Only differences between groups considered significant (*P* values < 0.05) are displayed.

The observation that circRNA activates innate immunity prompted the investigation of the capacity of circRNA to subsequently induce adaptive immune responses when used as an adjuvant and as an antigenic encoding sequence. We designed a circRNA-encoding chick Ovalbumin (hereafter named circOVA). To maximize circRNA translation, we used previously optimized elements for our circRNA design and transcription (12) (Fig. 3*A*). These elements include optimized RNA chemical modifications, 5′ and 3′ untranslated regions, internal ribosome entry sites (IRESs), and synthetic aptamers shown to increase circRNA translation over mRNA after a single transfection.

We first validated the protein production activity of circOVA in 293T cells. Twenty-four hours after circOVA transfection, Ovalbumin protein was detected in cell lysate by immunoblot (Fig. 3*B*) and in supernatant by ELISA (210 pg OVA/μL of supernatant).

We proceeded to validate the activation of adaptive immune responses after in vivo delivery of circRNA-encoding protein. The immunogenicity of circOVA alone was compared to soluble OVA protein combined with circRNA as an adjuvant (circRNA+ OVAp). Mice were intranasally immunized with either m6A-modified or -unmodified circOVA, and 30 d postboost, the lungs were analyzed for antigen-specific T cell responses. *N*6-methyladenosine (m6A) modification has been shown to promote the translation of circRNAs (9); however, we have previously shown that m6A abrogates circRNA immunity (18). Indeed, naked delivery of m6A-modified circOVA did not induce any OVA-specific T cell responses (*SI Appendix*, Fig. S4*A*). A subset of animals in the unmodified circOVA group generated potent OVA-specific CD8 T cell responses, but many other animals did not (*SI Appendix*, Fig. S4*A*). We suspect that after in vivo delivery of naked circOVA, the amount of circRNA that enters antigen-presenting cells is sufficient to induce immune signaling activation; however, only a small proportion of circRNA might be readily accessible for translation.

### CircRNA Delivery with CART Improves circRNA Translation.

We hypothesized that a delivery vehicle might be required to induce optimal immune responses in vivo with circRNA as both immunogen and adjuvant. We tested circRNA delivery using CARTs, a class of synthetic biodegradable materials shown to complex, protect, and efficiently deliver mRNA (36, 37) and circRNA (12) intracellularly, leading to highly efficient protein translation.

An essential process for initiating cytotoxic immune responses is antigen presentation (38). To determine whether circRNA delivered with CART (referred to as CART-circRNA) can be translated and processed for antigen presentation by dendritic cells, we tested the antigen presentation capacity of MutuDCs after transfection with CART-circOVA. We measured the capability of antigen-primed dendritic cells to induce antigen-specific T cell proliferation in vitro (Fig. 3*C*). Dose titration showed that only 0.1 ng circRNA is required to induce antigen-specific T cell proliferation when circRNA is complexed with CART (Fig. 3*D*). These observations indicate that the protein encoded by circRNA can be processed and presented to the immune system.

We also asked whether the recognition of circRNA and consequent activation of the innate immune system differs between naked and CART-mediated circRNA delivery. Mice were s.c. immunized with CART alone, naked circRNA, or CART-circRNA. Innate cell frequencies, activation, and circOVA uptake were measured in the inguinal LNs at 24 h following immunization. circRNA delivered with CART resulted in a similar activation profile of innate immune subsets as previously observed with naked circRNA (Fig. 3*E*). Immunization with CART alone induced some innate immunity, such as increased monocyte frequencies in the iLNs (*SI Appendix*, Fig. S5*A*), as well as increased CD86 expression on mDCs (Fig. 3*E*). However, delivery of circRNA with CART did not significantly alter circRNA recognition by immune cells (*SI Appendix*, Fig. S5*B*) or the innate cell infiltration and activation in the iLNs (*SI Appendix*, Fig. S5*A*). Thus, CART does not impact the recognition of circRNA by the innate immune system and subsequent activation of dendritic cells and macrophages.

### CircRNA-Encoding Antigen Induces Strong T Cell Responses In Vivo.

**Fig. 4.**
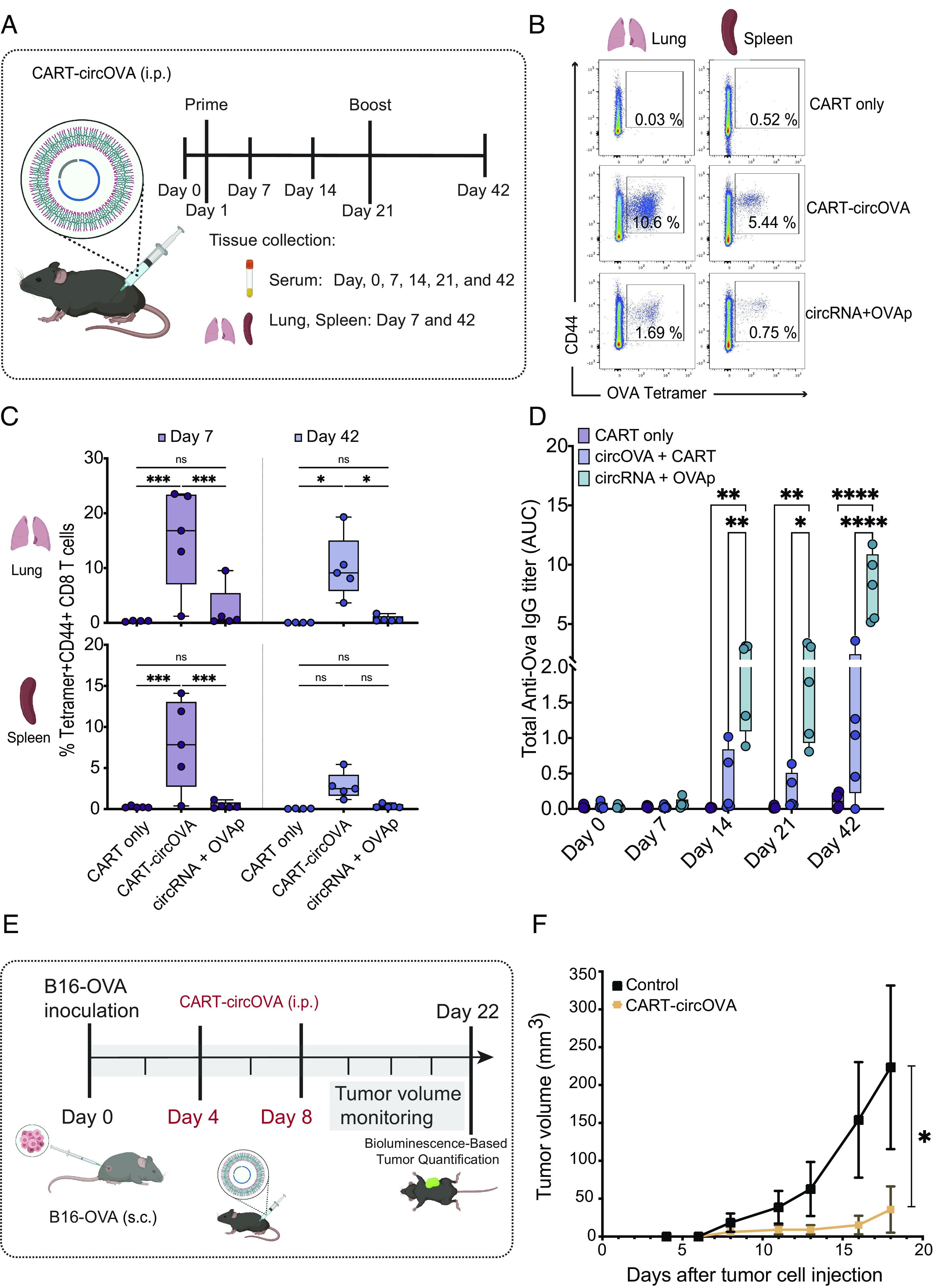
Circular RNA delivery in vivo activates T cell–specific responses. (*A*) Schematic representation of immunization strategy and monitoring of adaptive immune responses. Nine micrograms of circOVA was complexed with CART reagent and delivered intraperitoneally at days 0 and 21. Serum samples were collected weekly, and the spleen and lung were analyzed 7 d post prime and 21 d postboost. (*B*) Percentage of OVA-specific T cell responses in the lung and spleen at day 42 (representative sample). (*C*) Quantification of OVA-specific T cells in the lung and spleen at day 7 and day 42 (n = 5, bars represent Min and Max). (*D*) Time course analysis of anti-OVA IgG antibodies in serum measured by ELISA (n = 5, bars represent Min and Max). (*E*) Schematic representation of immunization strategy and monitoring of tumor volume after inoculation with B16-F10-OVA cells. Ten micrograms of circOVA was complexed with CART reagent and delivered intraperitoneally at days 4 and 8 after tumor cell inoculation. (*F*) Tumor volume monitoring over 22 d (n = 5, bars represent SEM). Results are representative of three independent experiments. Two-way ANOVA was applied in *C* and *D*. Repeated-measures ANOVA was applied in *F*. *P < 0.05, **P < 0.01, **P < 0.001, ****P < 0.0001. Differences between groups were considered significant for *P* values < 0.05. ns, not significant.

CARTs have been shown to work effectively in mice, have a high encapsulation efficiency, and are well tolerated and nonimmunogenic (36, 37, 39). We also previously showed robust and sustained protein production from circRNA delivered with CART after intraperitoneal injection (12). Thus, we selected to measure adaptive immune responses in mice after intraperitoneal delivery of circRNA-encoding protein. Three groups of mice were intraperitoneally immunized with either CART alone (vehicle-only control), CART-circOVA, and circRNA+OVAp at days 0 and 21. Antigen-specific CD8 T cell responses were assessed by MHC class I tetramer staining of the lung, spleen, and blood T cells at day 7 (7 d postprime) and day 42 (21 d postboost) (Fig. 4*A*).

We observed that CART-circOVA induced potent CD8 T cell responses in the lung, spleen (Fig. 4 *B* and *C*), and blood (*SI Appendix*, Fig. S6*B*) at 7 d following a single immunization. Three weeks after the booster immunization (day 42), significant levels of CD8 T cell responses are observed in the CART-circOVA group in the spleen and lung (Fig. 4 *B* and *C*), yielding approximately sixfold to eightfold greater frequency of OVA-specific CD8 T cells compared to circRNA+OVAp. Moreover, CART-circOVA immunization regimen induced both KLRG1+CD127- effector cells (short-lived effector cells or SLECs, ~20%) and KLRG1-CD127+ memory cells at day 42, with the latter T cell population being comprised heavily of effector memory T cells (TEM, ~35%) and a lower fraction of central memory (TCM) and resident memory (TRM) cells (*SI Appendix*, Fig. S6 *A* and *C*).

In addition, to measure the antibody responses following immunization, mice were bled at days 0, 7, 21 (preboost), and 42 (21 d postboost). We observed that while circRNA+OVAp induces consistent and significantly higher anti-OVA IgG compared to CART-circOVA, anti-OVA antibodies were still detectable in the CART-circOVA group (Fig. 4*D*). The lack of consistent high-titer antibody responses may result from a reduced protein secretion after CART-circOVA immunization. Consistent with this notion, we were not able to detect OVA protein in blood 24 h after immunization. These results indicate that our immunization strategy with CART-circRNA leads to a T cell biased response and further optimization may be required to induce strong antibody responses to circOVA.

We next sought to determine whether the combination of circRNA and CART could be generalized to additional vaccine candidates. Given that influenza virus is one of the most rapidly mutating viruses, strategies aimed at targeting the virus’s conserved regions have become crucial in the last few decades. In particular, CD8 T cells targeting conserved sites of the virus (such as nucleoprotein) have been shown to be protective against heterosubtypic influenza infection (40, 41), and T cell responses in humans have also been shown to correlate with protection (42). Therefore, we constructed a circRNA encoding the nucleoprotein (NP) sequence of the influenza virus (PR8 strain), referred to as circNP. Previous studies have shown that nucleoprotein-specific T cells are important in both homotypic and heterotypic protection against influenza virus infection (43–45), making NP-specific T cells a great target for a universal influenza vaccine. Intraperitoneal immunization with circNP + CART (CART-circNP) at days 0 and 21 resulted in the induction of nucleoprotein-specific T cell responses in the blood at day 7 postboost, as measured by class I tetramer staining of CD8 T cells (ASNENMETM epitope) (*SI Appendix*, Fig. S7 *A* and *B*). Our results indicate that using circRNA to encode pathogen-derived antigenic sequences can effectively induce antigen-specific CD8 T cell responses after immunization.

Our data suggest that synthetic circular RNAs can encode both the antigen and adjuvant activity required for immunization. Likewise, the route, dose, and manner of circRNA delivery impact the programmed immune response's potency, consistency, and memory.

### CircRNA Vaccine Induces Antitumor Efficacy.

Cancer vaccination aims to induce antigen-specific T cell–based cellular immunity capable of targeting and clearing tumor cells (46). The strong cytotoxic T cell responses observed systemically in tissue and blood after immunization with circOVA complexed with CART prompted us to further investigate circRNA as a cancer vaccine. We hypothesize that vaccine-induced OVA-specific T cells should eradicate OVA-expressing tumors. Moreover, the antitumor response should be systemic and be elicited by vaccination at a site distant from the tumor (i.e., abscopal effect). We tested the antitumor efficacy of the CART-circRNA vaccine in a therapeutic regime. C57BL/6 mice were randomly assigned into two groups: a control group (untreated) and a CART-circOVA (vaccine) group. Syngeneic B16-F10-OVA melanoma cells were inoculated subcutaneously on the backs of all mice. CART-circOVA formulations were injected intraperitoneally 4 and 8 d after tumor cell inoculation (Fig. 4*E*). The circRNA vaccine group showed a significant tumor growth inhibition compared to the untreated group (Fig. 4*F*). Bioluminescence imaging confirmed eradication of luciferase-labeled cancer cells (*SI Appendix*, Fig. S8 *A* and *B*). These results indicate that circRNA immunization could serve as an effective cancer immunotherapy to inhibit tumor growth in vivo.

## Discussion

The potential of circRNA as a vaccine platform and gene delivery system has gained substantial interest since the speedy development and FDA approval of the mRNA vaccine against SARS-CoV-2 (24). CircRNA is an attractive platform as biomarkers and vectors for gene expression due to its superior durability and stability. However, the extent to which extracellular circRNAs may engage the innate and adaptive immune systems is poorly understood.

In this study, we investigated the molecular and functional effects of circRNA recognition by the innate immune system. We elaborated on the potential of circRNA as an effective adjuvant when inoculated via various immunization routes. Our results showed that circRNAs, combined with protein antigens, can induce potent adaptive responses regardless of the immunization route. Notably, the mucosal immunization (intranasal delivery) of circRNA and OVAp induced potent lung-resident memory T cell responses. We observed the induction of cytosolic RNA sensors RIG-I and MDA5; inflammatory cytokines IL1-B, TNFa, and IL-6; and activation markers MHC-I and CD40 after circRNA uptake by dendritic cells. This phenotypic profile suggests that the uptake of circRNA by APCs is sufficient to induce a dendritic cell activation and maturation that enables them to interact with antigen-specific T cells. In vivo delivery of fluorescently labeled circRNA allowed us to characterize circRNA recognition by the innate immune system. We showed specific circRNA response by monocytes, macrophages, and dendritic cells in draining lymph nodes after subcutaneous delivery of naked circRNA. Significant proliferation of B cell, monocyte, and macrophage populations was observed 24 h following immunization with circRNA. In addition, all macrophages and dendritic cell subsets found in draining lymph nodes were shown to be activated based on the surface expression of CD86. Similarly, we observed a fast increase in proinflammatory cytokines and chemokines CCL5, CCL4, and CCL7, which act as chemoattractants of macrophages, T cell subsets, and DCs, among other immune cell types. In combination, these results highlight the potential of circRNA to induce proliferation and enhanced recruitment of specialized immune cell subsets, leading to the initiation of effective adaptive immunity.

We were able to combine the dual roles of exogenous circRNAs as an adjuvant and a template for antigen expression, showing that circRNAs can serve as delivery systems and as immune potentiators. Our in vitro experiments in the MutuDC cell line showed that circOVA could be translated, and peptide sequences were efficiently loaded onto the MHC class I proteins for presentation to T cells, resulting in the activation and proliferation of antigen-specific T cells. To examine the capacity of circRNA to induce adaptive immunity in vivo, we immunized mice with circOVA complexed with CART. We observed potent and persistent T cell responses in mice following immunization. Interestingly, antibody responses were not as effectively induced by the current CART-circOVA formulation, likely due to limited expression of cell-free soluble OVA protein vs. the intracellular pool. However, recent work has shown that T cell–inducing vaccines can provide protective immunity against simian–HIV (SHIV) even when suboptimal antibody responses are present (25, 26). Nonetheless, future studies of the underlying mechanisms responsible for the induction of T cell responses without efficient induction of antibody responses are warranted. And, induction of more potent antibody responses will be important for infectious disease applications of circRNA vaccination.

Taken together, we demonstrated the potential of circRNAs to generate an acute inflammatory environment that favors the generation of potent cellular immunity. Combining the features of facile programmability, durable antigen expression, and safe administration, the use of circRNAs as a tool to program antigen-specific T cell response has the potential to advance prophylactic or therapeutic vaccines for infectious diseases and cancer immunotherapy.

## Materials and Methods

### CircRNA Design and In Vitro Transcription.

CircRNA templates were synthesized by cloning DNA fragments into a custom entry vector which contains self-splicing introns, 5′ PABP spacer, HBA1 3′ UTR, and HRV-B3 IRES. CircRNA was synthesized using HiScribe T7 High-Yield RNA Synthesis Kit (NEB E2040S). IVT templates were PCR amplified (Q5 Hot Start High-Fidelity 2x Master Mix) and column purified (Zymo DNA Clean & Concentrator-100) prior to RNA synthesis as previously described (12). Briefly, 1 µg circRNA PCR-template was used per 20 µL IVT reaction. Reactions were incubated overnight at 37°C. The IVT templates were subsequently degraded with 2 µL DnaseI (NWB M0303S) for 20 min at 37°C. The remaining RNA was column purified and digested with 1U RnaseR per microgram of RNA for 60 min at 37°C. Samples were then column purified, quantified using a Nanodrop One spectrophotometer, and verified for complete digestion using an Agilent TapeStation. CircRNA was fluorescently labeled by incorporating 5% of Fluorescein-12-UTP (Sigma-Aldrich 11427857910) in the corresponding IVT reaction, or by posttranscriptional modification using Label IT Nucleic Acid Labeling Kit (Mirus Bio Cy3, Cy5, Fluorescein, or AF488). In all experiments, we used a mixture of unlabeled circRNA and fluorescently labeled circRNA at 20:1 ratio. Three circRNAs were produced: circOVA which encodes OVA protein, circNP which encodes influenza nucleoprotein, and circFOR that has a frame-shift sequence that interferes with protein translation. CircOVA and circNP were enhanced for translation by adding 5% m6A modifications (when specified) and 5% of 2′OMeC for in vivo delivery. Circular RNA elements and modifications are listed in *SI Appendix*, Table S1.

### Cell Lines.

MutuDC cells were purchased from Applied Biological Materials Inc. (ABM T0528). The cells were maintained in IMDM-Glutamax (Gibco 31980) medium supplemented with 10% FBS, 1% penicillin-streptomycin, 10 mM Hepes (Gibco 15630), and 50 μM β-mercaptoethanol (GIBCO 31350). B16 murine melanoma cell line and HEK293 cells were obtained from ATCC and cultured in DMEM medium supplemented with 10% FBS and 1% penicillin/streptomycin (Thermo Fisher).

For routine subculture, 0.25% Trypsin-EDTA (Thermo Fisher) was used for cell dissociation. All cell lines were kept in culture at 37°C in a humidified incubator with 5% CO2 and regularly tested for *Mycoplasma* contamination (Lonza LT07-318).

### T Cell Proliferation Assay.

OT-I CD8 T cells were purified from TCR-transgenic mice OT-I by negative selection using immunomagnetic beads (Miltenyi Biotech). For direct MHC-I antigen presentation assays, MutuDC lines were seeded at 10,000 cells per well in round-bottom 96-well plates. For MHC-I-restricted antigen presentation assays, MutuDC cells were incubated for 2 h with 1 nM SIINFEKL (OVA257–264, Sigma-Aldrich S7951), 1 mg/mL Ovalbumin protein (InvivoGen vac-pova), 1 μg circFOR, or 1 μg circOVA, in the presence or absence of 1 μM CpG (ODN 1585, InvivoGen). The cells were washed three times in medium and incubated with 50,000 purified CFSE-labeled OT-I CD8 T cells (CellTrace CFSE Cell Proliferation Kit, Invitrogen C34554). T cell proliferation was measured after 60 h of culture by flow cytometry analysis excluding doublets and dead cells. OT-I CD8 T cells were gated as CD8+ Vα2+ cells. Live dividing T cells were detected as low for cell proliferation dyes (CFSE low). MutuDC cells were similarly transfected with circOVA with or without CART reagent at the indicated concentrations.

### qRT-PCR Measurement of Immune Receptors.

MutuDC cells were seeded as previously described and treated with 1 μM CpG or 1 μg circRNA in serum-free media. Twenty-four hours after treatment, total RNA was isolated from cells using TRIzol (Invitrogen, 15596018) and Direct-zol RNA Miniprep (Zymo Research, R2052) with on-column DNase I digestion, following the manufacturer’s instructions. RT-qPCR analysis was performed in triplicate using Brilliant II SYBR Green qRT-PCR Master Mix (Agilent, 600825) and a LightCycler 480 (Roche). The relative RNA level was calculated by the ddCt method compared to B-Actin control. Primer sequences are listed in *SI Appendix*, Table S2.

### Flow Cytometry Analysis of Cytokines and Surface Receptors.

MutuDC cells were seeded as previously described and treated with 1 μM CpG or 1 μg circRNA in serum-free media. Twenty-four hours after treatment, cell supernatant was collected and the cytokine levels were quantified using the cytometric bead array kit for mouse inflammatory cytokines (BD Cytometric Bead Array (CBA) Mouse Inflammation Kit). Similarly, cell suspensions were transferred to a v-bottom plate and washed twice with PBS, stained with Live/dead NIR fixable dye, and stained with anti-MHC-II (redFluor 710 Tonbo 80-5321-U025), anti-MCH-I (PE, eBioscience 12-5958-82), anti-CD86 (APCFire/750, BioLegene 105045), anti-CD40 (PerCP-eFluor 710, eBioscience 46-0401-80), and anti-CD80 (Pe-cy5, eBioscience 15-0801-82). After 30-min incubation on ice, the cells were then washed and analyzed by flow cytometry.

### circOVA Protein Measurements.

8 x105 293T cells were transfected with 5 μg circOVA using TransIT-mRNA transfection kit (Mirus MIR 2250), with 3 µL TransIT-mRNA reagent (Mirus Bio) per microgram of RNA. Twenty-four hours after transfection, the cells were collected and lysed to extract total proteins. Bioruptor sonication with RIPA buffer (150 mM NaCl, 1% Triton X-100, 0.5% sodium deoxycholate, 0.1% SDS, 50 mM Tris, pH 8.0) was used to lyse the cells. Proteins were fractionated by sodium dodecyl sulfate-polyacrylamide gel electrophoresis (SDS-PAGE), transferred to nitrocellulose membranes, blocked in phosphate-buffered saline containing 4% (wt/vol) nonfat milk for 1 h rocking at room temperature, and then incubated overnight at 4°C with the indicated primary antibody. OVA was validated with 1:500 Ovalbumin polyclonal antibody (Novus Biologicals, NB600-922-0.1 mg), and antialpha tubulin antibody was used as loading control (Abcam, ab7192). Secondary antibodies were incubated for 1 h at 1:15,000, in pairs depending on the primary antibody identities: IRDye 800CW Goat anti-Mouse IgG (Li-COR Biosciences, 926-32210) and IRDye 680RD Goat anti-Rabbit IgG (Li-COR Biosciences, 926-68071). Western blot detection and quantification was done using an Odyssey infrared imaging system (Li-COR Biosciences). Similarly, OVA concentrations in cell culture supernatants were measured 24 h transfection with Ovalbumin (OVA) ELISA Kit (Abbexa, abx259051).

### Mice and Immunizations.

Wild-type C57BL/6J (000664) mice were purchased from Jackson Laboratories. The mice were matched for sex and aged between 8 and 14 wk. For immunization, the mice were injected intranasally with 30 μL circRNA (25 μg per mouse), intravenously with 100 μL circRNA (25 μg per mouse), subcutaneously at the base of the tail with 100 μL circRNA (25 or 50 μg per mouse when indicated), and intraperitoneally with 100 μL CART-circOVA (9 μg per mouse). When indicated, 50 ug Ovalbumin protein (InvivoGen vac-pova) was also delivered in combination with 25 μg Poly(I:C) (HMW VacciGrade, InvivoGen vac-pic) or 50 μL AddaVax (InvivoGen) as per the manufacturer’s instructions (each mouse should receive 50 μL AddaVax if performing subcutaneous injections). The approved institutional animal care and use committee (IACUC) protocols of Stanford University were followed when handling all the mice.

### Flow Cytometry Analysis of Innate Immune Subsets.

Draining inguinal lymph nodes from immunized mice were collected and treated as previously described (47, 48). Briefly, the tissues were treated with collagenase type IV (Worthington) at a concentration of 1 mg/mL for 20 min at a temperature of 37 °C, and then passed through a 100-μm strainer to obtain a single-cell suspension. The resulting single-cell samples were stained with a range of markers including Zombie UV (BUV496, BioLegend 423107), anti-Ly6C (BV780, BioLegend 128041), anti-Ly6G (APC-Cy7, BioLegend 127624), anti-CD19 (BUV395, BD 563557), anti-CD3 (BB700, BD742175), anti-MHCII (AF700, eBioscience 56-5321-82), anti-CD11b (BV650, BioLegend 101239), anti-CD11c (BV421, BioLegend 117330), anti-CD86 (A647, BioLegend 105020), anti-Siglec-F (PE-CF594, BD 562757), anti-CD45 (BV610, BioLegend 103140), anti-CD169 (PE-Cy7, BioLegend 142412), anti-PDCA-1 (BUV563, BD 749275), anti-CD8a (BUV805, BD 612898), anti-CD103 (PE, eBioscience 12-1031-82), anti-NK1.1 (BV510, BioLegend 108738), and anti-F4/80 (BUV737, BD 749283). The cells were analyzed using the BD FACSymphony analyzer located at the Stanford Shared Fluorescence-activated cell sorting (FACS) Facility.

### CD8 T Cell Flow Cytometry Analysis.

Whole lung, spleen, or peripheral blood from immunized mice was collected after the indicated time points. The lung and spleen were digested with collagenase type IV (Worthington) at a concentration of 1 mg/mL for 20 min at a temperature of 37 °C, and then passed through a 100-μm strainer to obtain a single-cell suspension. Red blood cells were lysed before staining. Single-cell samples were then stained with Zombie Yellow (BUV570, BioLegend 423103), anti-CD3 (clone 145-2C11, BioLegend), anti-CD8α (clone 53-6.7, BioLegend), anti-CD4 (clone RM4-5, BioLegend), anti-CD44 (clone IM7, BioLegend), anti-CD45 (clone 30-F11, BioLegend), anti-CD69 (clone H1.2F3, BioLegend), anti-CD103 (clone 2E7, BioLegend), and Ova-specific tetramer (residues 257 to 264). The MHC class I tetramers used in this study (H-2K(b)-SIINFEKL and H-2D(b)-ASNENMETM) were kindly provided by the NIH Tetramer Core Facility. Cells were analyzed with an LSRII.UV analyzer at the Stanford Shared FACS Facility.

### Antibody ELISA.

At the designated time points, serum was collected from the immunized mice. Ovalbumin (OVA) protein was procured from InvivoGen and used to coat high-binding 96-well plates at a concentration of 10 µg/mL in PBS. The plates were blocked with TBS containing 2% BSA and washed before adding serially diluted serum samples, which were then incubated at 37 °C for 1 h. After washing the wells three times with TBS and 0.05% Tween-20, secondary HRP-tagged goat anti-mouse IgG and IgA (SouthernBiotech, 1:5,000 dilution) was added, and the wells were incubated for another hour at 37 °C. The wells were washed three times before the addition of 3,3′,5,5′-tetramethylbenzidine substrate solution (Thermo Pierce). The reaction was stopped after 5 min with 0.16 M sulfuric acid. Finally, the optical density at 450 nm was measured with a SpectraMax Microplate Reader. The reciprocal EC50 and end point titers were calculated by GraphPad Prism.

### Luminex Assay.

This assay was performed by the Human Immune Monitoring Center at Stanford University as previously described (47). Mouse 48 plex Procarta kit (Thermo-Fisher/Life Technologies) was used according to the manufacturer's instructions but with some modifications. The samples were added to a plate containing beads that were linked with antibodies and incubated overnight at 4°C with shaking. Then, a biotinylated detection antibody was added for 60 min at room temperature with shaking. After washing the plate, streptavidin-PE was added for 30 min at room temperature, and the plate was washed again. Reading buffer was added to the wells, and each sample was measured in duplicate. The plates were read using a Luminex 200 or a FM3D FlexMap instrument with a lower bound of 50 beads per sample per cytokine. Custom Assay Chex control beads were added to all wells.

### CART Synthesis and circRNA Complex.

O6-stat-N6:A9 CARTs (here referred simply as CART) consist of a block of on average 12 subunits made up of a statistical 1:1 mixture of oleyl- (O) and nonenyl-substituted (N) carbonate subunits followed by a block of on average 9 α-amino ester subunits (A). CircRNA formulation with CART was prepared as previously described (36, 37) CircRNA was diluted in PBS pH 5.5 and mixed with CART at 1:10 charge ratio immediately before in vitro transfection in MutuDC or intraperitoneal delivery into mice.

### Mouse Model of Subcutaneous Melanoma.

B16-F10-OVA cells were harvested for injection in PBS at 1 × 106 cells/mL. One hundred microliters of cell suspension (1 × 105 cells/mouse) was subcutaneously injected into C57BL/6 mice. The mice were monitored daily for tumor incidence and growth. When palpable, tumors were measured every other day using digital calipers and measured in two dimensions. Tumor volume (V) was determined by using the formula V = L × W × D × 3.14/6. The mice were killed before the tumors became necrotic in the center.

### Statistical Analysis.

Prism software version 9.2.0 was used to perform statistical analyses. One-way and two-way ANOVA tests were used to compare more than two groups, and differences between groups with *P*-values below 0.05 were considered significant. Sample sizes were not predetermined using statistical methods, and mice were randomly assigned to experimental groups. Data collection and analysis were not blinded to the conditions of the experiments.

## Supplementary Material

Appendix 01 (PDF)

## Data Availability

All study data are included in the article and/or *SI Appendix*.
