# Supplementary material for: Circular RNA vaccine induces potent T cell responses
Source: Proc Natl Acad Sci U S A. 2023 May 8;120(20):e2302191120. doi: 10.1073/pnas.2302191120 (PMC10193964; doi:10.1073/pnas.2302191120)
Supplement: Supplementary file 1 — Appendix 01 (PDF) [file pnas.2302191120.sapp.pdf]

Supporting Information for

Circular RNA vaccine induces potent T cell responses

Laura Amaya[1,2,*], Lilit Grigoryan[3,*], Zhijian Li[4], Audrey Lee[3], Paul A. Wender[4,5], Bali Pulendran[3,†], Howard Y. Chang[1,6,†]

[1] Center for Personal Dynamic Regulomes, Stanford University, Stanford, CA 94305, USA.
[2] Institute for Stem Cell Biology and Regenerative Medicine, Stanford University School of Medicine, Stanford, CA 94305, USA.
[3] Institute for Immunity, Transplantation and Infection, Stanford University, Stanford, CA 94305, USA.
[4] Department of Chemistry, Stanford University, Stanford, CA 94305, USA.
[5] Department of Chemical and Systems Biology, Stanford University, Stanford, CA 94305, USA
[6] Howard Hughes Medical Institute, Stanford University, Stanford, CA 94305, USA.
[*] These authors contributed equally.
[†]Correspondence to: H.Y.C. at howchang@stanford.edu and B.P. bpulend@stanford.edu

This PDF file includes:

Figures S1 to S8
Tables S1 to S2

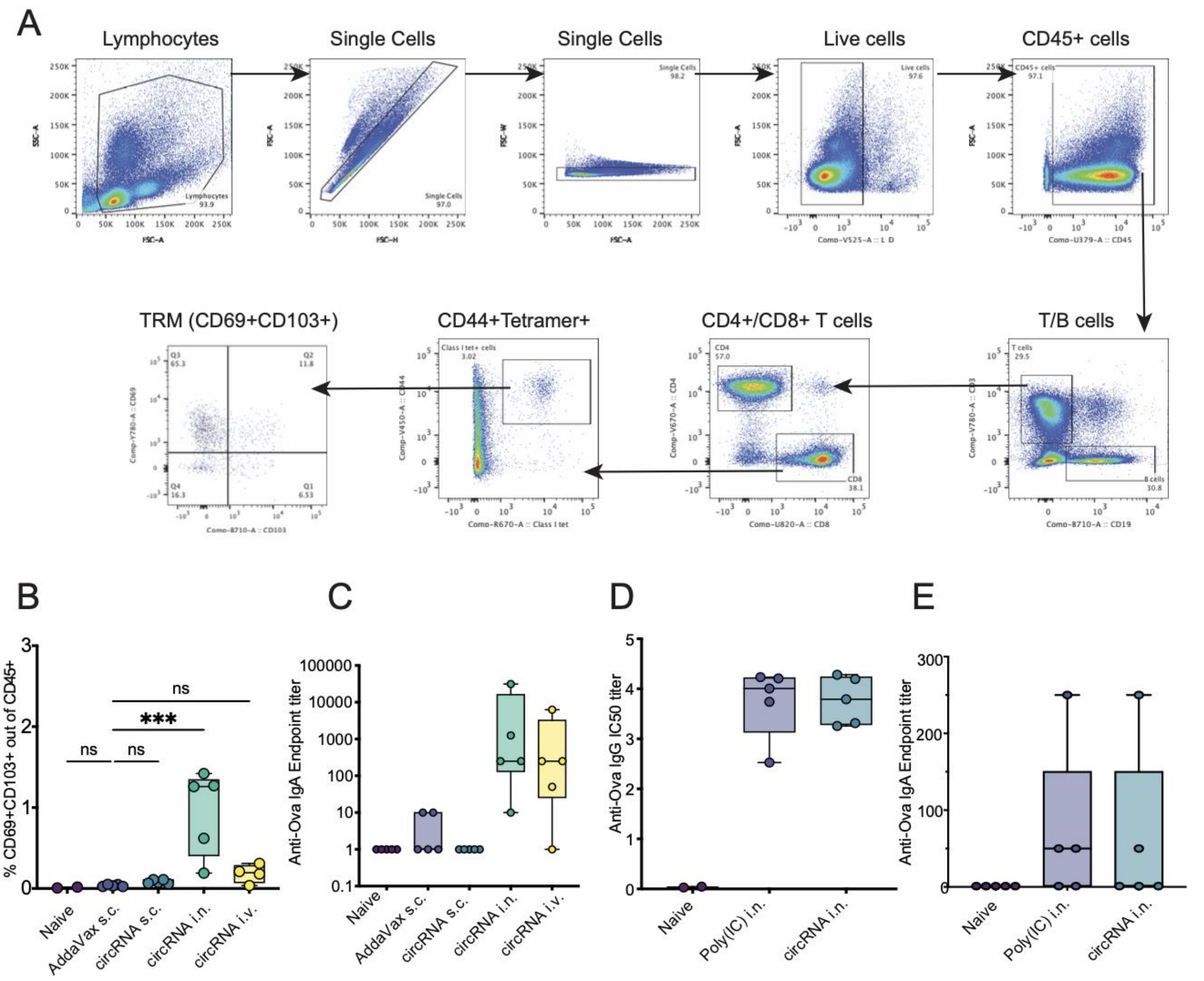

Supplementary Fig. 1. Adjuvant effect of circRNA by different routes of delivery. (A) Gating strategy used to distinguish adaptive immune subsets after delivery of circRNA. (B) Percentage of OVA-specific TRM cells (gated as CD69+CD103+) in lung as % of CD45+ live cells 30 post-boost after circRNA immunization by different delivery (n = 5, bars represent Min and Max). (C) IgA antibodies in serum measured by ELISA at day 30 postboost after circRNA immunization by different delivery routes (n = 5, bars represent Min and Max). (D) anti-OVA IgG (reciprocal EC50 titers shown) (E) and IgA antibodies (endpoint titer shown) in serum measured by ELISA at day 30 postboost of i.n. delivery of circRNA compared to Poly(IC) (n = 5, bars represent Min and Max). One-way ANOVA was applied in B-E. $^*P < 0.05$, $^{**}P < 0.01$, $^{***}P < 0.001$, $^{****}P < 0.0001$. Differences between groups were considered significant for P values < 0.05. ns, not significant.

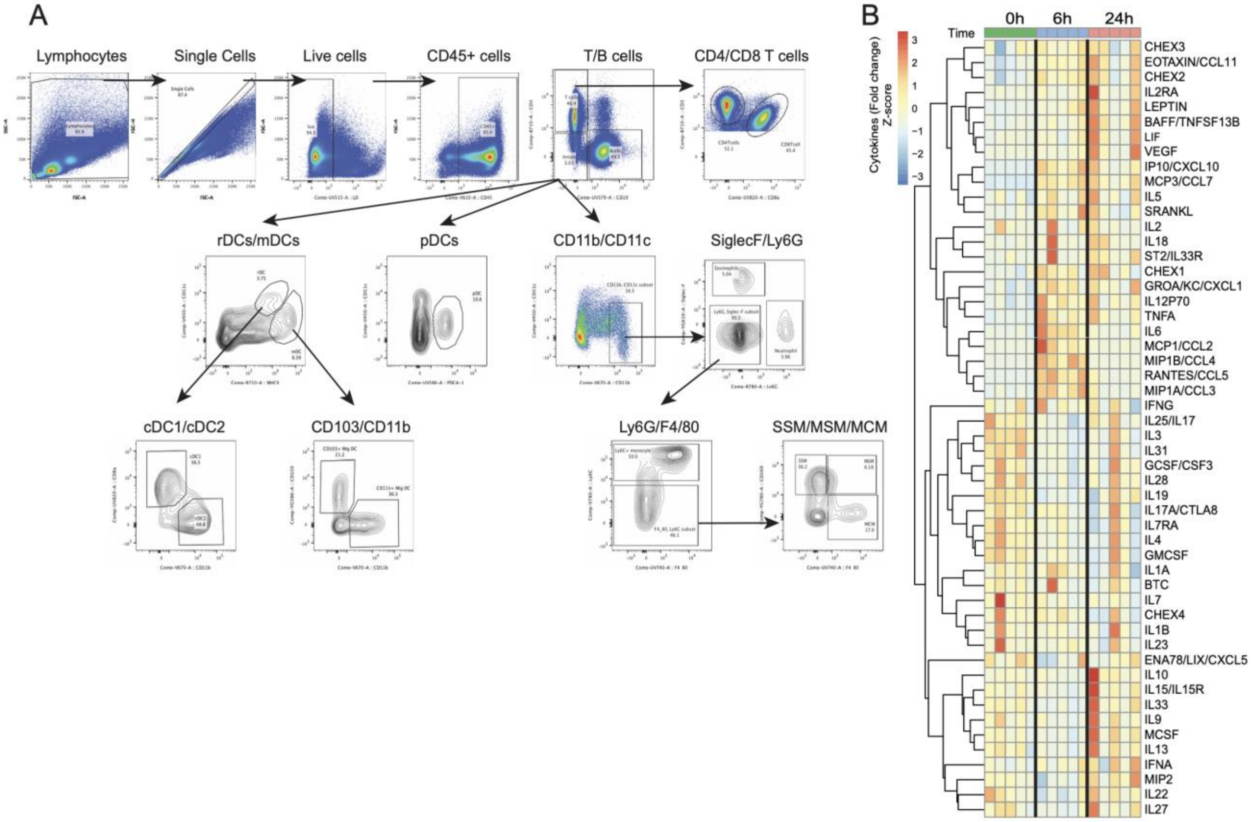

Supplementary Fig. 2. Innate immune response after delivery of circRNA in vivo. (A) Gating strategy used to distinguish innate immune subsets after s.c. delivery of circRNA. (B) Complete panel of cytokines measured by Luminex Assay at 6 and 24 hours after s.c. delivery of circRNA.

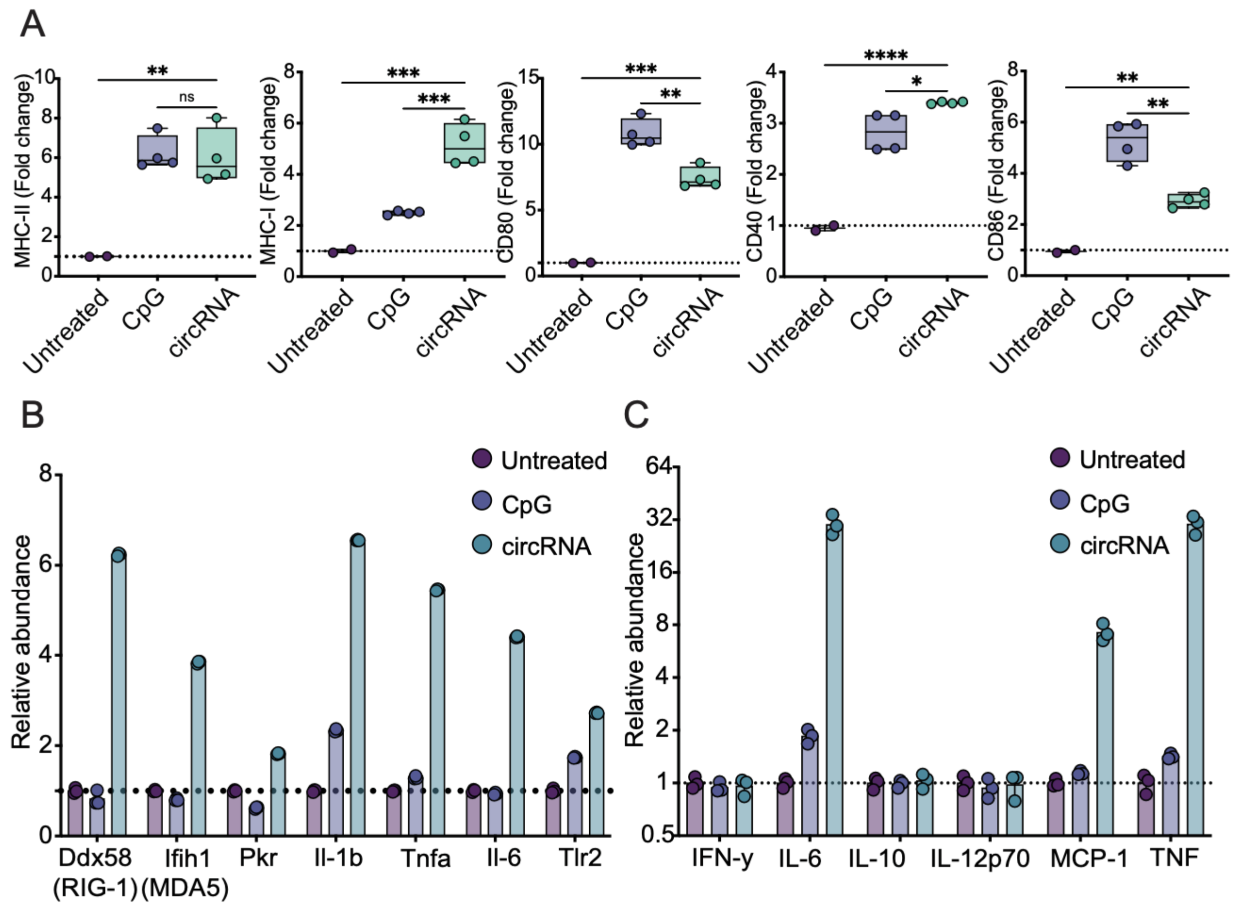

Supplementary Fig. 3. circRNA induces DC maturation and activation. (A) Flow cytometry quantification of activation markers after MutuDC cells incubation with circRNA compared to CpG (n = 4, bars represent Min and Max). (B) qRT-PCR quantification of immune receptors and cytokines and (C) flow cytometry quantification of secreted inflammatory cytokines after MutuDC cells incubation with circOVA compared to CpG (n = 3, bars represent SD). One-way ANOVA was applied in A. **P* < 0.05, ***P* < 0.01, ****P*< 0.001, *****P* < 0.0001. Differences between groups were considered significant for *P* values < 0.05. ns, not significant.

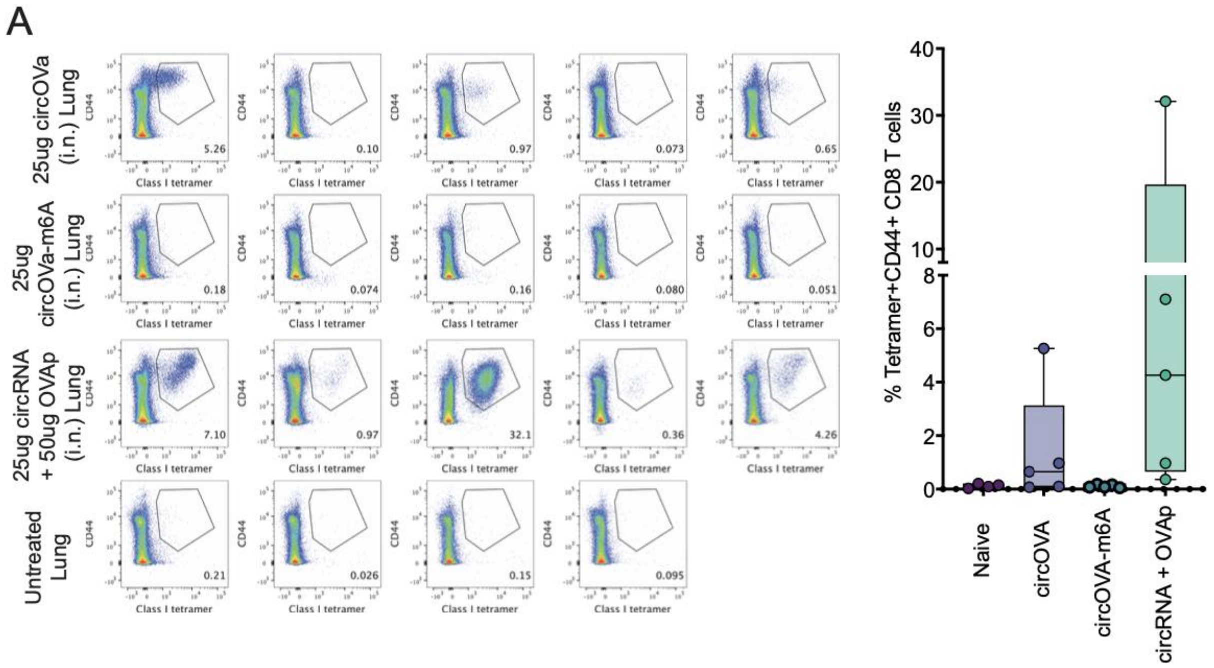

Supplementary Fig. 4. Adaptive immune responses after immunization with circOVA. (A) Percentage of OVA-specific T cell in lung after i.n. immunization with naked circOVA, circOVA modified with m6A, or circRNA + OVAp. Individual CD44+Tetramer+ gates and summary bar graph are displayed on the right (n = 5, bars represent Min and Max).

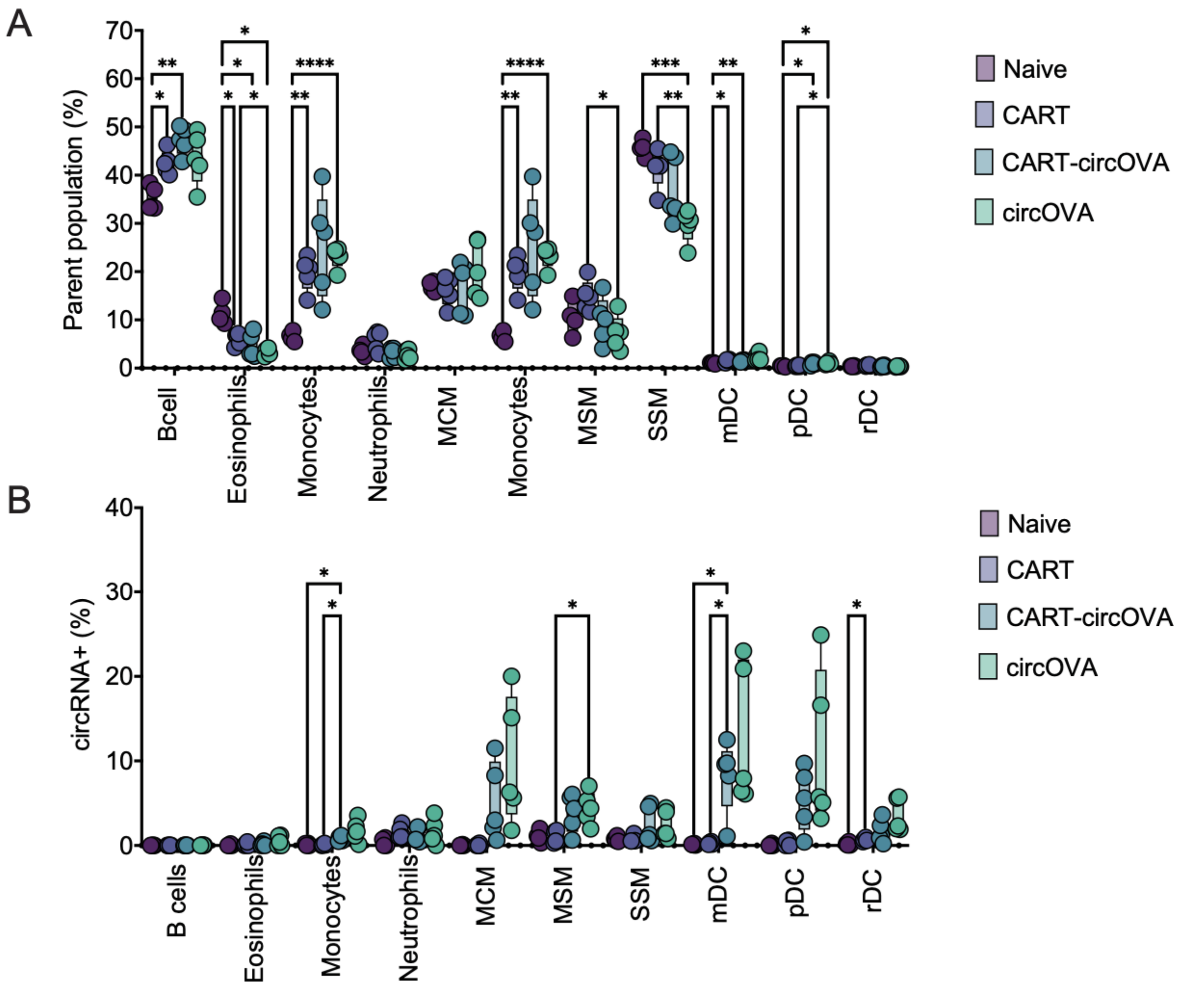

Supplementary Fig. 5. Innate immune response to circRNA immunization with and without CART. (A) Frequencies of innate cell subsets in the draining inguinal LNs and (B) circRNA+ cells as % of given cell subsets 24 h after s.c. delivery of circRNA, circRNA delivered with CART (CART-circOVA), and CART alone. (n = 5, bars represent Min and Max). Two-way ANOVA was applied $^*P < 0.05$, $^{**}P < 0.01$, $^{***}P < 0.001$, $^{****}P < 0.0001$. Only differences between groups considered significant (P values < 0.05) are displayed.

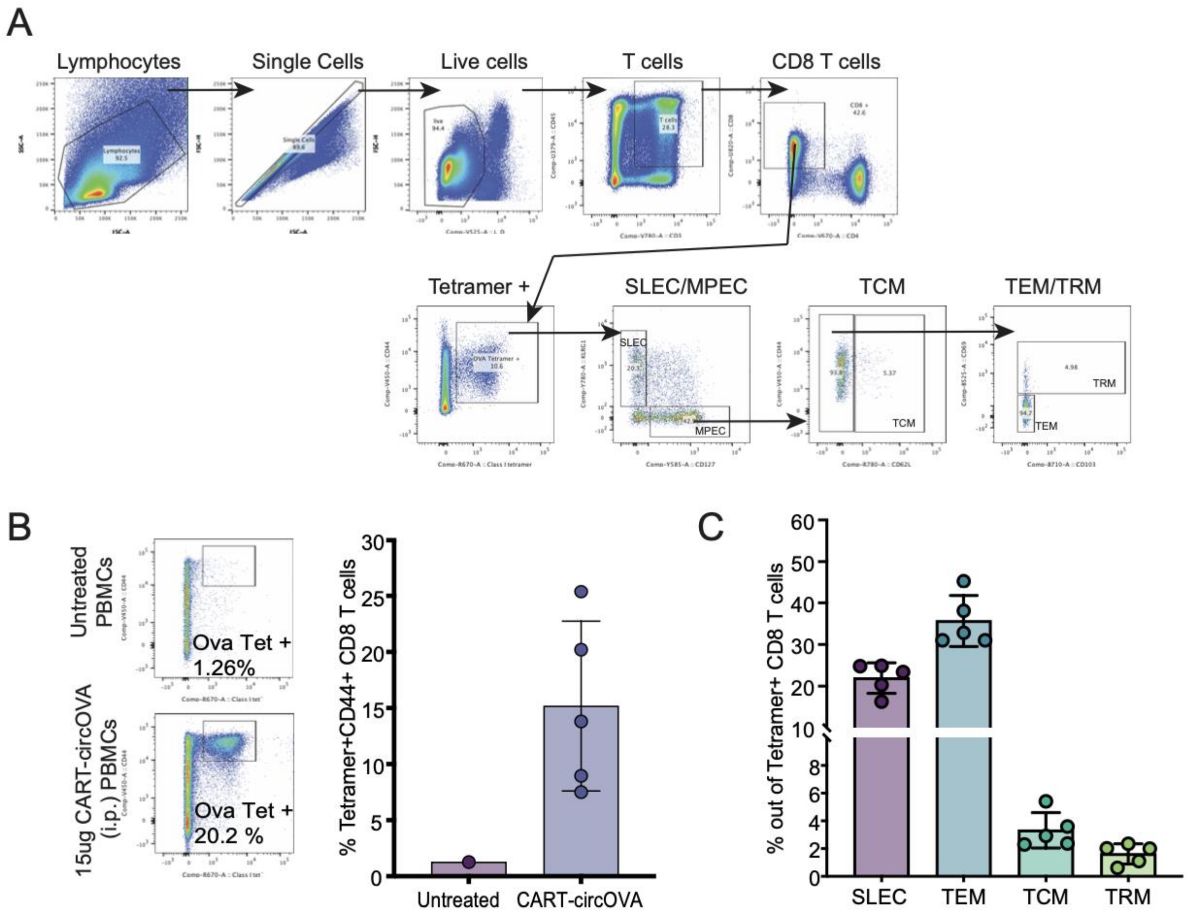

Supplementary Fig. 6. Adaptive immune responses after immunization with CART-circOVA. (A) Gating strategy used through immunization experiments to measure antigen specific T cell responses and various T cell effector subsets. (B) Percentage of OVA-specific T cells in peripheral blood at day 7 post-prime (n = 4, bars represent SD). (C) Proportion of SLEC, TEM, TCM (Central memory T cells) and TRM subsets at day 21 post-boost (n = 5, bars represent SD).

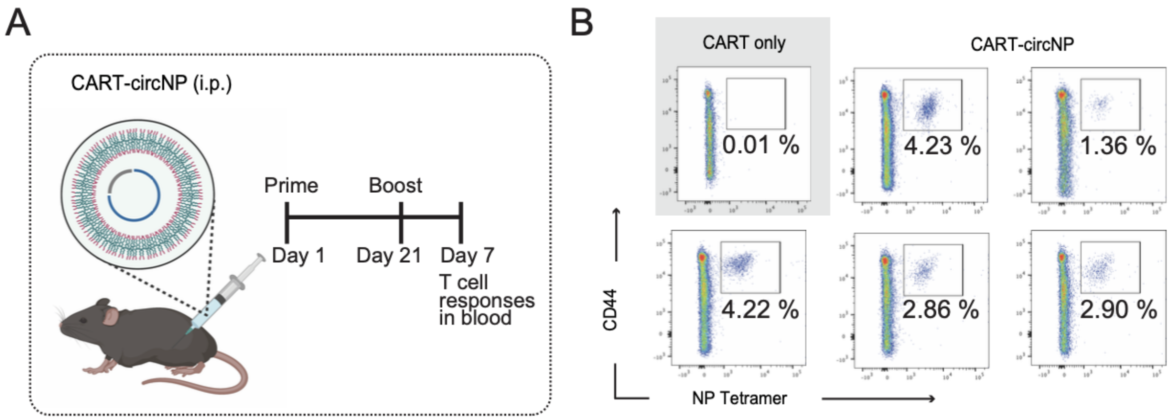

Supplementary Fig. 7. Antigen-specific T cell responses after immunization with CART-circNP. (A) Experimental design of CART-circNP immunization and monitoring of T cell responses in blood 7 days postboost. (B) Percentage of OVA-specific T cell in blood after i.p. immunization with CART-circNP, displaying individual CD44+Tetramer+ gates.

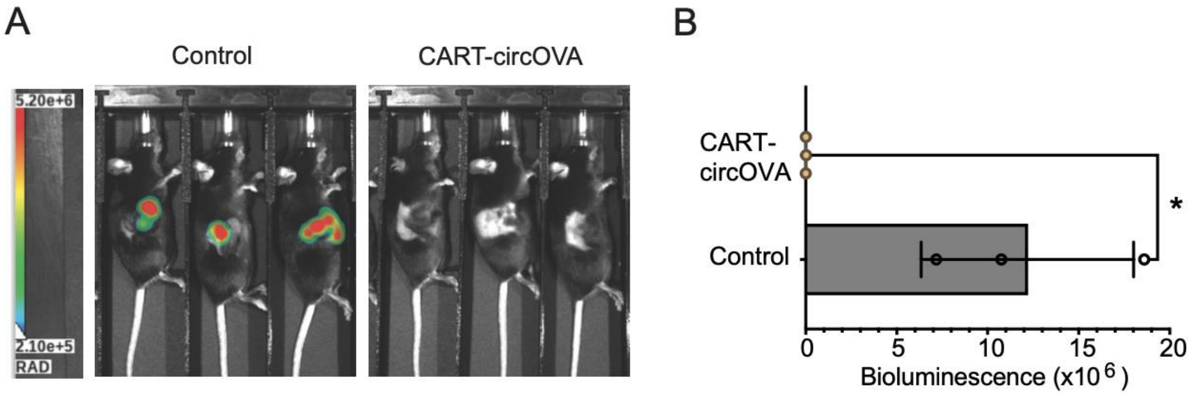

Supplementary Fig. 8. Adaptive immune responses after immunization with CART-circOVA. (A) Representative bioluminescence images and (B) quantification of tumors from control mice and mice vaccinated with circOVA. (n = 3, bars represent SD). Unpaired t-test was applied *$P < 0.05$.

Table S1. List of circRNAs used throughout experiments.

Figure	circRNA	CDS	RNA modification	IRES	5'UTR	3'UTR	Labeling
**Figure 1**	circForeign	GFP (out of frame)	none	iCVB3	none	none	none
**Figure 2**	circForeign	GFP (out of frame)	none	iCVB3	none	none	AF488
**Figure 3**	circOVA	Ovalbumin	5% 2'-O-MethylIC	synIRES-RC25 (iHRVB3-based)	5′ PABP spacer	HBA1	none
**Figure 4**	circOVA	Ovalbumin	5% 2'-O-MethylIC	synIRES-RC25 (iHRVB3-based)	5′ PABP spacer	HBA1	none
**Supplementary Figure 1**	circForeign	GFP (out of frame)	none	iCVB3	none	none	none
**Supplementary Figure 2**	circForeign	GFP (out of frame)	none	iCVB3	none	none	AF488
**Supplementary Figure 3**	circForeign	GFP (out of frame)	none	iCVB3	none	none	none
**Supplementary Figure 4**	circOVA	Ovalbumin	5% m6A	synIRES-RC25 (iHRVB3-based)	5′ PABP spacer	HBA1	none
**Supplementary Figure 4**	circOVA	Ovalbumin	none	synIRES-RC25 (iHRVB3-based)	5′ PABP spacer	HBA1	none
**Supplementary Figure 4**	circForeign	GFP (out of frame)	none	iCVB3	none	none	none
**Supplementary Figure 5**	circOVA	Ovalbumin	5% 2'-O-MethylIC	synIRES-RC25 (iHRVB3-based)	5′ PABP spacer	HBA1	AF488
**Supplementary Figure 6**	circOVA	Ovalbumin	5% 2'-O-MethylIC	synIRES-RC25 (iHRVB3-based)	5′ PABP spacer	HBA1	none
**Supplementary Figure 7**	circNP	Influenza Nucleoprotein	5% 2'-O-MethylIC	synIRES-RC25 (iHRVB3-based)	5′ PABP spacer	HBA1	None
**Supplementary Figure 8**	circOVA	Ovalbumin	5% 2'-O-MethylIC	synIRES-RC25 (iHRVB3-based)	5′ PABP spacer	HBA1	none

Table S2. Primer list used for qRT-PCR measurement of immune receptors.

Primer name	Sequence
qRTPCR-mus-RIGI-R	CAG ATC CGA GAC ACT AAA GGG A
qRTPCR-mus-RIGI-F	TCC TCA TCA GCC TTG CTT TCA
qRTPCR-mus-MDA5-F	ATG GAC GCA GAT GTT CGT GG
qRTPCR-mus-MDA5-R	TCC CTT CTC GAA GCA AGT GTC
qRTPCR-mus-PKR-F	ATG CAC GGA GTA GCC ATT ACG
qRTPCR-mus-PKR-R	TGA CAA TCC ACC TTG TTT TCG T
qRTPCR-mus-IL1B-F	AAG GGC TGC TTC CAA ACC TTT GAC
qRTPCR-mus-IL1B-R	ATA CTG CCT GCC TGA AGC TCT TGT
qRTPCR-mus-TNF-A-F	TCT CAT GCA CCA CCA TCA AGG ACT
qRTPCR-mus-TNF-A-R	ACC ACT CTC CCT TTG CAG AAC TCA
qRTPCR-mus-IL6-F	TCCAGTTGCCTTCTTGGGAC
qRTPCR-mus-IL6-R	GTACTCCAGAAGACCAGAGG
qRTPCR-mus-TLR2-F	ACAGCAAGGTCTTCCTGGTTCC
qRTPCR-mus-TLR2-R	GCTCCCTTACAGGCTGAGTTCT
qRTPCR-mus-B-ACT-F	GAC TAC CTC ATG AAG ATC CTG ACC
qRTPCR-mus-B-ACT-R	CTC AGT AAC AGT CCG CCT AGA AG
